# Accounting for Diffusion in Agent Based Models of Reaction-Diffusion Systems with Application to Cytoskeletal Diffusion

**DOI:** 10.1371/journal.pone.0025306

**Published:** 2011-09-23

**Authors:** Mohammad Azimi, Yousef Jamali, Mohammad R. K. Mofrad

**Affiliations:** Molecular Cell Biomechanics Laboratory, Department of Bioengineering, University of California, Berkeley, California, United States of America; The University of Akron, United States of America

## Abstract

Diffusion plays a key role in many biochemical reaction systems seen in nature. Scenarios where diffusion behavior is critical can be seen in the cell and subcellular compartments where molecular crowding limits the interaction between particles. We investigate the application of a computational method for modeling the diffusion of molecules and macromolecules in three-dimensional solutions using agent based modeling. This method allows for realistic modeling of a system of particles with different properties such as size, diffusion coefficients, and affinity as well as the environment properties such as viscosity and geometry. Simulations using these movement probabilities yield behavior that mimics natural diffusion. Using this modeling framework, we simulate the effects of molecular crowding on effective diffusion and have validated the results of our model using Langevin dynamics simulations and note that they are in good agreement with previous experimental data. Furthermore, we investigate an extension of this framework where single discrete cells can contain multiple particles of varying size in an effort to highlight errors that can arise from discretization that lead to the unnatural behavior of particles undergoing diffusion. Subsequently, we explore various algorithms that differ in how they handle the movement of multiple particles per cell and suggest an algorithm that properly accommodates multiple particles of various sizes per cell that can replicate the natural behavior of these particles diffusing. Finally, we use the present modeling framework to investigate the effect of structural geometry on the directionality of diffusion in the cell cytoskeleton with the observation that parallel orientation in the structural geometry of actin filaments of filopodia and the branched structure of lamellipodia can give directionality to diffusion at the filopodia-lamellipodia interface.

## Introduction

Diffusion is a key driver of many biological processes in living systems where ions and molecules move down concentration gradients as a result of their thermal motion within solutions. This phenomenon can be modeled using various computational techniques that consume varying degrees of computational resources correlated with the degree of molecular detail provided by the model. Of specific interest are modeling techniques that account for diffusion and reaction of molecules in biological systems.

Current methods for modeling reaction-diffusion systems generally rely on ordinary differential equation (ODE) models in which the system is assumed to be well-mixed and molecules of interest exist in high numbers, satisfying the continuum assumption [1]–[3]. These models ignore both the spatial detail and the stochastic behavior observed in natural systems. Other techniques with applications to modeling cellular pathways include partial differential equation (PDE), chemical master equation (CME) and reaction-diffusion master equation (RDME) models that are capable of accounting for spatial varying levels of spatial detail and stochasticity at the cost of increased computational time. These techniques are well-suited for modeling a range of biological phenomena (ODE/PDE methods are ideal for metabolic network models, CME/RDME methods are ideal for gene expression models), with each technique limited by spatial, stochastic and computational cost constraints [1]–[5]. On the other end of the modeling spectrum are more accurate Brownian dynamics (BD) and Langevin dynamics (LD) models that explicitly account for the diffusion and interaction of individual molecules with the ability to track these individual molecules and assess the effects of spatial and environmental properties that result in the emergence of phenomena such as molecular crowding. These models have additional computational costs associated with them, resulting in limitations to the simulation time and length scales. Recently, agent based models (ABM) have been applied to simulating reaction-diffusion systems [6]–[9] and have the potential to bridge the gap between spatiotemporally detailed but computationally expensive BD/LD methods and the less detailed but computationally inexpensive ODE/PDE/CME/RDME methods.

### Agent Based Models

Agent based modeling is a robust computational technique used to simulate the spatiotemporal actions and interactions of real-world entities, referred to as “agents” in an effort to extract their combined effect on the system as a whole. Both space and time are discretized in an agent based model, giving these autonomous agents the ability to move and interact with other agents and their environment at each time step over a given duration. Simple behavioral rules govern the movement and interaction of each individual entity in an effort to re-create or predict more complex behavior of multiple entities. Such a model attempts to simulate the emergence of complex phenomena that may not be apparent when simply considering individual entities. Agent based modeling has seen applications in a broad range of fields ranging from artificial intelligence and gaming to modeling emergent social behavior such as the spread of disease and outcomes of financial markets [10]–[14]. In their simplest form, these agent based models consist of a mesh of “cells” that make up the discretized space that agents occupy. The agents occupy these cells and are typically only aware of other agents within their “neighborhood”; in the simplest form a neighborhood consists of adjacent cells. Agents are given the ability to move into adjacent cells and to interact with other agents with some probability in conjunction with governing rules that define what movement and interactions are possible.

In a physical system we can attribute the diffusion of a particle in solvent to the instantaneous imbalance of the combined forces exerted by collisions of the particle with the much smaller solvent molecules surrounding it which are moving due to random thermal motion. In an agent based model the same movement of this particle due to collisions with much smaller solvent molecules can be implicitly modeled by correlating the diffusion coefficient of the particle in the specific solvent to some movement probability for that particle. Furthermore, in a physical system, steric effects prevent two particles from coming closer than a certain distance from one another or occupying the same position. This type of behavior can also be replicated with an agent based model using governing rules that limit the number of particles per discretized space. As a result of these simplifications, the process of modeling particles diffusing throughout a space does not require computationally intensive method for simultaneously calculating velocities of particles and the effects of repulsive and attractive forces of these particles on other particles within the system (as seen in BD/LD models). Rather, we can describe diffusion and interaction in terms of natural language based on simple observations such as: *different particles move throughout space in a random manner*, *these movements are related to particle size*, and *two particles tend to disfavor occupying the same space*. These descriptions based on natural language can be translated into simple logic rules that govern the behavior of the system. Although ABMs seem ideal for modeling reaction-diffusion systems, existing ABM frameworks do not consider the accuracy of particle movement algorithms. Furthermore, particle movement probabilities are oftentimes selected arbitrarily by the modeler without validating that the molecules' movement behavior represents realistic diffusion rates. Subsequently, agent based modeling of biochemical systems can benefit from validated movement algorithms and movement probability selection criteria.

We have outlined an approach for establishing the logic rules that govern particle diffusion along with methods for translating key parameters such as diffusion coefficients that have continuous and deterministic values into probabilities that can be used as inputs to a discrete and stochastic agent based model. Additionally, we validate these methods with single-particle and multi-particle simulations where normal diffusion is modeled. Furthermore, we investigate the effects of molecular crowding and high concentrations of macromolecules in the simulation volume as is seen in the cell cytoplasm along with their effect on effective diffusion coefficients, comparing our results with Brownian dynamics simulations. We then investigate the effect of allowing multiple particles to reside in a discrete cell of finite volume and quantify and discuss advantages and disadvantages of various approaches of enforcing finite cell volumes. Finally, we apply the ABM framework to investigate the role of geometry on the directionality of diffusion and show how specific geometries can promote diffusion in a particular direction while other geometries hinder the movement of macromolecules in a particular direction as seen in the filopodia and lamellipodia regions of the cell cytoplasm with regard to the diffusion of G-actin.

## Results and Discussion

### Relating Diffusion Coefficients to ABM Movement Probabilities

Given a lattice with discretization length of *ΔL*, and dimensionality of *N_Dimension_* (either 1, 2 or 3), the relationship between diffusion coefficient (*D*) and movement probability (*T*) for a fixed timestep of *Δt* is shown in Eq. (1) (see Methods for derivation).
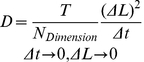
(1)


The relationship established in Eq. (1) allows us to take diffusion coefficient values that are meaningful in a continuous and deterministic framework and apply them to a discrete and stochastic agent based model via movement probabilities. The relationship between mean square displacement and time can be used to validate the relationship derived in Eq. (1) for simulating diffusion via ABM. This means that movement probability associated with the diffusion coefficient being modeled should result in displacement behavior and rate that would be seen in a physical system. The mean square displacement 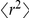 of a particle diffusing due to Brownian motion is proportional to the time elapsed through the following relationship [15]:
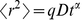
(2)


Where *q* is the numerical constant which depends on dimensionality, *q* = 2, 4 or 6 for dimensionalities of 1, 2 or 3 respectively and *D* is the diffusion coefficient and *t* is time. The exponent α is the anomalous diffusion exponent where α = 1 for normal diffusion while all other values of α represent anomalous diffusion. This means that for normal diffusion, there is a linear relationship between the mean square displacement of a particle and time. If we were to plot the calculated mean square displacement versus time in our simulation, the linearity of this plot would demonstrate whether simulated diffusion is normal or anomalous and the slope of this plot would be related to our diffusion coefficient as described by Eq. (2).

To validate our model for the diffusion of a single particle, we simulated a macromolecule with a Stokes radius of *r = 5 nm* (*diameter = 10 nm*) that was free to diffuse in a solvent in three dimensional space with a diffusion coefficient of *D = 100 µm^2^/s*. We ran our model for 500,000 time steps with a minimum sampling size of 300 independent runs which resulted in a linear relationship between mean square displacement and time (*α = 0.9992, R^2^ = 0.9999*) which implies that the model successfully reproduces normal diffusion behavior. Furthermore, when modeling multiple particles with different diffusion coefficients in very low concentration (crowding effects are negligible), it was observed that using Eq. (1) to derive movement probabilities for each particle type produced the same linear relationship between the measured mean square displacement and time (normal diffusion was observed) with different slopes for each particle type that corresponded to the diffusion coefficients being modeled. The deviation from linearity in this case was on the same order as that of the single particle and is most attributed to the stochastic nature of the model and the sampling size used. Additionally, it should be noted that as Δt and ΔL become larger (more coarse grained models) the error in the simulation also increases as a result of the approximations made in Eq. (1). However, this discretization error is typically negligible when compared to the variations resulting from the stochasticity of the model and more importantly, such a change in discretization will result in the loss of detailed spatial and temporal information.

### Crowding effects on Diffusion and Multiparticle Occupation of Cells

The relationship between crowding due to increased concentration and the effective diffusion at low time scales is shown in Eq. (3) (see Methods for derivation).
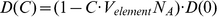
(3)


**Figure 1:**
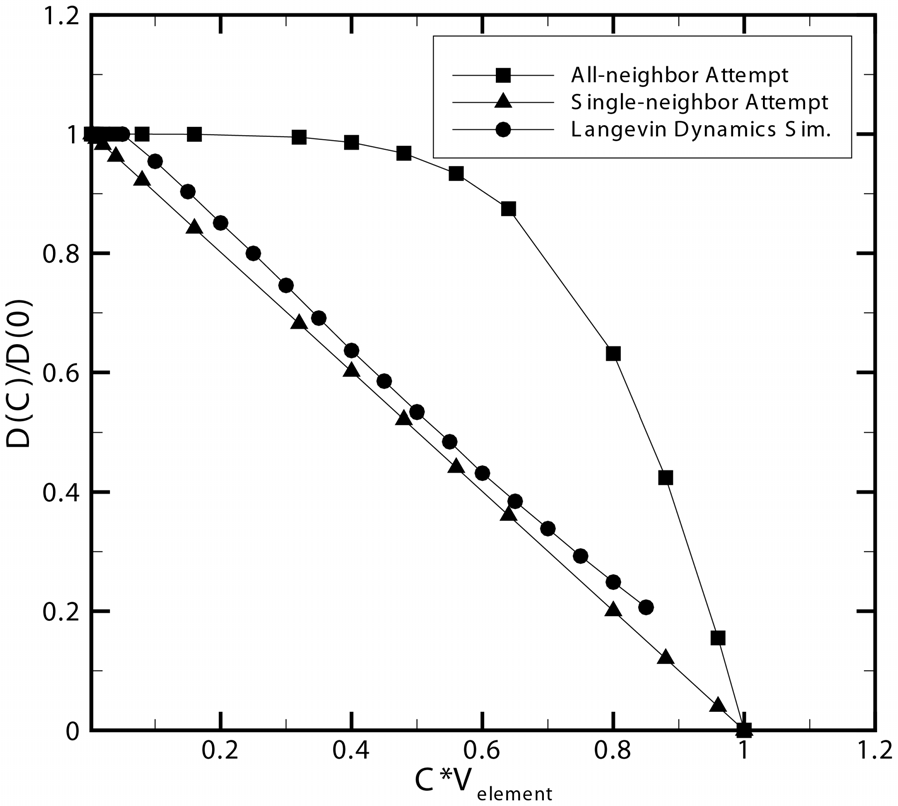
Comparison of algorithms for diffusion in crowded environments. Effective diffusion coefficient at low time scales versus normalized free particle concentration (volume density) for two agent based model algorithms and a Langevin dynamics simulation for comparison. The graph shows the single-neighbor attempt algorithm to best represent diffusion at higher concentrations as the effective diffusion of this algorithm decreases linearly with increased concentration as does the Langevin dynamics model. As the graph shows, the single-neighbor attempt and Langevin dynamics simulation exhibit the same negative linear slope with a slight difference in offsets resulting from the different definition of particle volume between the two modeling techniques. Higher concentration data points for Langevin dynamics have been omitted as the volume definition of particles leads to volume overlap at this concentration.

Where *D(C)* is the effective diffusion coefficient as a function of concentration and *D(0)* is the diffusion coefficient of a particle in a low concentration system and *C*, *V_element_* and *N_A_* represent concentration of crowding molecule, volume of a discrete element and Avagadro's number respectively and the product of these three terms is equivalent to the probability of finding any discrete cell to be occupied by a molecule (*P^occ^ = CV_element_N_A_*). In order to determine how higher concentrations affect particle diffusion we performed Langevin dynamics simulations utilizing the shifted force form of the Lennard-Jones potential energy function that assessed the effective diffusion coefficient of a particle as the concentration of particles in the system was increased. The analytical relationship shown in Eq. (3) is in agreement with the computational result from Langevin dynamics simulations shown in Fig. 1 (circle points). These results can be compared with the result of the two different diffusion algorithms, all-neighbor attempt and single-neighbor attempt, used in the agent based model as shown in Fig. 1 (square and triangular points respectively). The single-neighbor attempt algorithm results are in agreement with both the Langevin dynamics simulation as well as the analytical relationship, showing that as concentration of particles in the system increases, the effective diffusion coefficient decreases linearly. The all-neighbor attempt algorithm that searches for neighboring vacant cells results in unnaturally higher effective diffusion coefficients.

**Figure 2:**
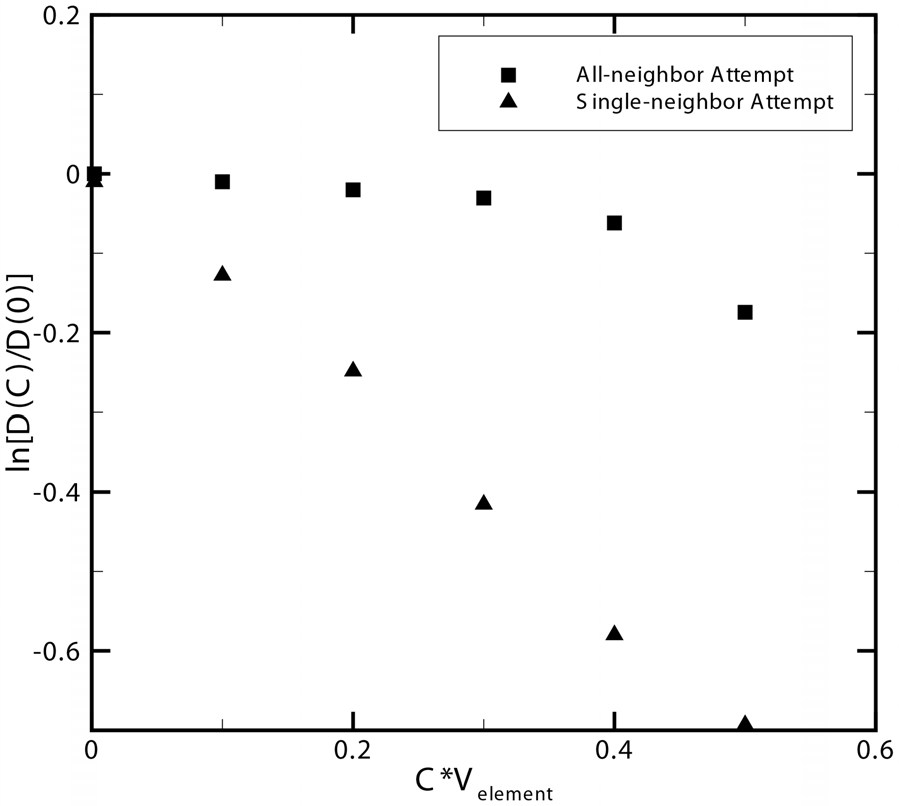
Natural log of anomalous diffusion of the Single-Neighbor attempt is in agreement with experimental data. Natural log of effective anomalous diffusion coefficient versus normalized free particle concentration (volume density) for two agent based model algorithms. The linear relationship between natural log of the anomalous diffusion coefficient and concentration in the single-neighbor attempt algorithm is in good agreement with experimental data for protein self diffusion [16].

The higher effective diffusion coefficient of the all-neighbor attempt algorithm can be attributed to the algorithm simulating “intelligent particles” that search for vacancies rather than the behavior of “non-intelligent particles” that diffuse randomly due to Brownian motion. The behavior exhibited by the single-neighbor attempt algorithm is considered best suited for modeling the diffusion of passive “non-intelligent” molecules and macro-molecules such as proteins involved in reaction-diffusion systems. Furthermore, this phenomenon is critical for modeling macromolecular crowding and its direct effects on intracellular diffusion as well as reaction kinetics in intracellular environments. Alternatively, the all-neighbor attempt algorithm would be better suited for intelligent agents that can sense the environment around them using means other than collisions. The implementation of a single-neighbor movement algorithm is a very computationally efficient way of providing detailed spatial information for diffusing particles while enforcing steric repulsion and simulating molecular crowding. It should be noted that both the agent based and Langevin dynamics methods in this comparison neglect conservation of momentum and energy as well as hydrodynamic interactions between particles. Nevertheless, ABM simulations using the single-neighbor attempt show a linear relationship between the natural log of the effective anomalous diffusion coefficient and concentration as shown in Fig. 2, which is in good agreement with experimental data despite neglecting hydrodynamic interactions [16]. Other methods such as stochastic rotation dynamics (SRD) are momentum and energy conserving and account for hydrodynamic interactions between particles in meso-scale simulations, but have not yet to date been applied to modeling reaction-diffusion systems [17].

The issue of crowding effects becomes more complex when considering systems with particles of varying size. In the simplest case where the model allows for only a single particle per cell, discretization errors can arise from small molecules saturating the available vacancies and reducing the effective diffusion coefficient, when in reality the volume density of the system has not been changed significantly. This error arises from discretization and the simplification that the smallest particles occupy the same volume as that of the largest particles. This issue can be overcome by introducing an additional layer of complexity in the agent based model where multiple particles are allowed to occupy a single cell. In this framework each particle is given a volume value, typically a fraction of the discretized cell's volume which it occupies, ranging from 0 to 1. As this framework is adopted, multiple particles are allowed to diffuse into a single cell and steric repulsion between particles is no longer intrinsically observed as it was with single particles per cell, raising concerns about individual cells' volume limits being exceeded at high concentrations of particles.

**Figure 3:**
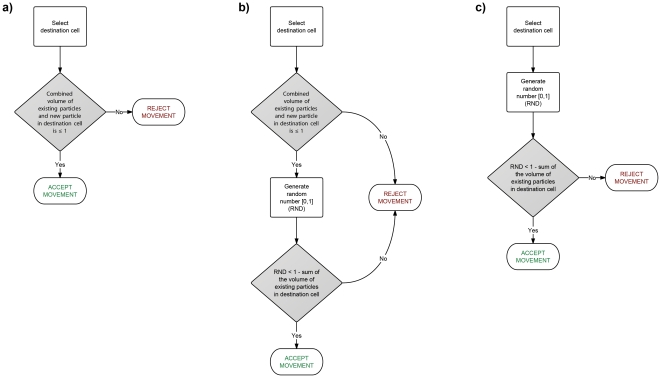
Algorithms for multi-particle per cell diffusion. Flowcharts showing the algorithm of three various methods for simulating steric repulsion of multiple particles per cell. a) the Volume Limit (VL) method is the most computationally efficient, b) followed by the Reduced Probability + Volume Limit (RP + VL) method and c) the Reduced Probability (RP) method being the least computationally efficient. The degree of accuracy for which each method models steric repulsion is illustrated in Fig. 4.

**Figure 4:**
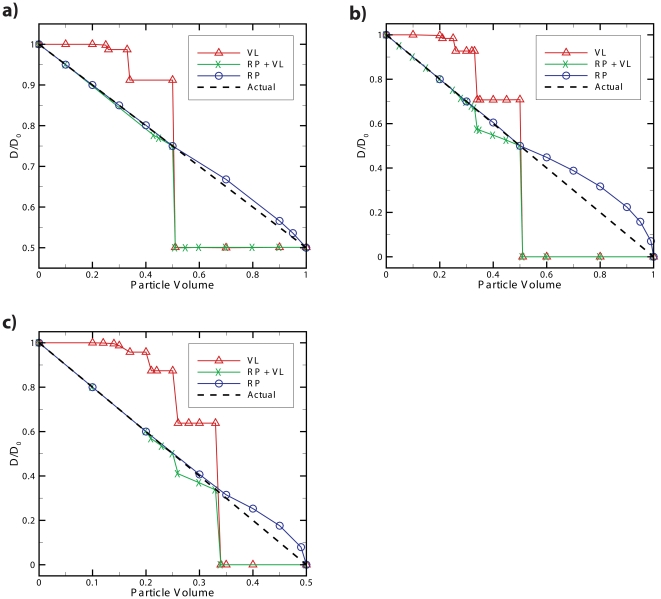
Comparison of the validity of multi-particle per cell diffusion algorithms. In systems of high concentration a) 500 particles in a system with 1000 cells, b) 1000 particles in a system with 1000 cells and c) 2000 particles in a system with 1000 cells, it can be seen that three different methods for handling the movement of multiple particles per cell result in significantly different behavior. The volume limit method (VL) is the most computationally efficient by simply limiting the movement of particles that would result in the fraction of occupied volume of a cell exceeding 1. However, it is also the least accurate when dealing with crowded environments. The combined reduced probability and volume limit method (RP + VL) is slightly less computationally efficient but is much more representative of crowded diffusion when the particles are of smaller volume. The reduced probability method (RP) is the least computationally efficient of the three but best represents the crowded diffusion for most particle sizes. Additionally, the system with 500 particles in 1000 cells deviates the least from actual when using the RP method while the more crowded systems deviate more, confirming that the RP method can accurately model physiologically relevant concentrations.

The most intuitive method for ensuring that the number of particles per cell does not exceed the cell's volume is to simply enforce that the movement of any particle into a destination cell will not surpass that cell's volume limit. Although this method is seemingly straightforward and adds minimal computational cost (see flowchart, Fig. 3.a), it results in the emergence of artificially high diffusion for particles of smaller size and artificially lower diffusion for larger particles (see Fig. 4). In addition, as Fig. 4 depicts, the effective diffusion of particles obeying the volume limit (VL) method is subject to artificial limitations resulting in the stair-step behavior. For example, in a concentrated environment where the cell's fraction volume cannot exceed 1, no cell can contain more than a single particle of volume fraction greater than 0.5. This means that when a three dimensional discretized space of 1000 elements has 1000 particles of size 0.51 fractional volume or larger (Fig. 4b), no particles in the system will diffuse since any movement will result in the volume limit being exceeded.

One method for rectifying the problem of artificially higher diffusion for smaller particles in the volume limit method is by adding a probability term based on cell capacity to the movement logic. In the combined reduced probability and volume limit method (RP + VL) shown in Fig. 4, as a cell's occupied volume increases, the probability of movement into that cell decreases. This adds additional computational time due to the random number generation required each time the cell's volume is not exceeded and a move is attempted (see flowchart, Fig. 3.b) but has the added benefit that it better matches the true behavior of the diffusion of multiple particles at physiologically relevant concentrations. However, as depicted in Fig. 4, this method of reduced probability combined with the volume limit is only effective at accurately modeling concentrated systems with smaller particles. Finally, we can best match the actual diffusion behavior by removing the volume limit and simply reducing the probability of movement based on the fraction of a cell's occupied volume (RP method shown in Fig. 4). This method is the most computationally intensive of the three as it requires a random number generation for every attempted move, regardless of whether a cell is occupied or empty (see flowchart, Fig. 3.c). This method is the suggested method when investigating systems with molecular crowding as it best conforms to the expected behavior of multiple particles in a concentrated environment which can be attributed to the steric repulsion that would prevent multiple particles from occupying the same position in space at a particular time. The error at high concentrations with larger particle sizes in the reduced probability method (RP) models each cell as an elastic box capable of briefly exceeding the cell's maximum volume. However, as shown in Fig. 4a, the error in this method is less than 5% at physiologically relevant crowding volumes of 10% - 40% excluded volume [18], [19].

### Geometry Effects on Diffusion

To demonstrate a biolgoical application of the proposed agent based diffusion method, we have modeled the effect of structural geometry on diffusion directionality. In this model, we show the effect of quasi-random versus parallel structural geometries of filaments similar to what is seen in the structure of cell actin filaments in the form of lamellipodia versus filopodia [20], [21]. Actin dynamics are thought to play a key role in cell motility [22]–[25]. Additionally, it has been shown that the flow of actin monomers in the lamellipodia cannot be explained by diffusion alone and may involve some form of active transport [26]. Moreover, due to the parallel orientation of actin filaments in the filopodia, and their longer length as a result of inhibition of the capping process, the actin monomers required for polymerization of actin filaments of the filopodia must travel a greater distance to where they are needed [21], . In this model, we investigate how the structural geometry and orientation of these filaments affects the directionality of diffusion of the monomers.

**Figure 5:**
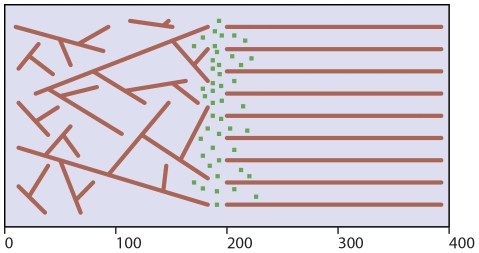
Representative cross-sectional illustration of simulation environment. Representatitve cross-sectional illustration of the xy-plane of the three dimensional simulation box of size *L_X_ = 400 nm, L_Y_ = 200 nm,* and *L_Z_ = 100 nm* with periodic boundary conditions in the y-direction only. The parallel filaments in the right half of the box (*x>200 nm*) represent the filopodia while filaments in the left half (*x<200 nm*) represent the lamellipodia in the cell. The green particles represent the freely diffusing actin monomers which are distributed in three dimensional space near *x = 200 nm*.

The model environment consists of a simulation box of size *L_X_ = 400nm, L_Y_ = 200nm,* and *L_Z_ = 100nm* with periodic boundary conditions in the y-direction only. Fig. 5 shows an illustration of a representative cross-sectional snapshot of the xy-plane of the simulation box with the right half containing parallel filaments and the left half containing a uniform density of filaments oriented at 68±2 degrees from one another in three dimensional space (prior to discretization) [20], [25], [30], [31]. This configuration was chosen not only to investigate geometry effects on diffusion but more specifically to model actin dynamics at the lamellipodia and filopodia interface.

**Figure 6:**
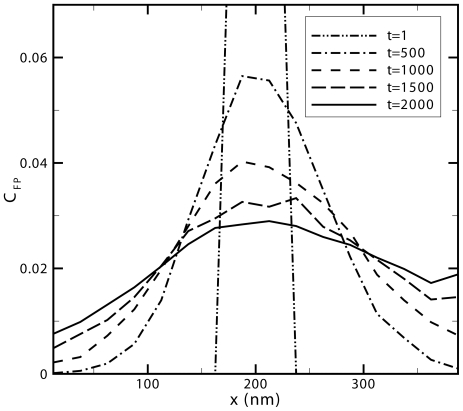
Density of diffusing actin monomers as a function of time and position. Normalized free particle concentration as a function of position for snapshots of time ranging from 1 to 2000 time steps with a time step increment of 74 µs for a fixed actin filament volume density of 0.25 averaged over ten runs. Initially at t = 1 the distribution of particles is uniform whereas at each subsequent time step shown, the filopodia region (*x>200nm*) is seen to have a higher free particle concentration than the lamellipodia region (*x<200nm*).

Actin filaments were initially generated in a non-discretized 3D environment with continuous filaments spaced a uniform distance apart and oriented parallel to one another within the filopodia region and conversely, filaments positioned randomly, with uniform density and oriented 68±2 degrees from each other in the lamellipodia region. The continuous actin polymers were then discretized into individual particles representing pairs of g-actin monomers fixed in space that occupy the full volume of each cell. It should be noted that the total number of particles (g-actin monomer pairs) in the lamellipodia and filopodia are equal to avoid obstacle concentration effects. Agent based modeling was used to investigate the effect of various actin filament densities on the directionality of free actin diffusion. As Fig. 6 illustrates, free particles diffuse more easily in the direction of the filopodia (*x>200 nm*) as opposed to the direction of the lamellipodia (*x<200 nm*). The simulation was run using characteristic values for the g-actin monomer diameter and diffusion coefficient of *ΔL = 5nm* and *D = 5.65 µm^2^/s* [21], [32], [33] in a three dimensional space, *N_Dimension_ = 3* with a movement probability of *T = 1* given that only one particle type is diffusing in the simulation and the movement probability is maximized in order to maximize computational efficiency. Subsequently, the time step of the simulation can be determined to be *Δt = 74 µs* using the relationship established in Eq. (1).

In order to show the time progression of concentration differences between the filopodia region and lamellipodia region at different fixed actin filament volume densities, we calculate the ratio of the center of mass of diffusing particles in the filopodia region to that of the lamellipodia. Eq. (4) shows the method used for calculating the center of mass for each region where *R* represents the center of mass and *N_i_* represents the number of freely diffusing actin monomers at position *x_i_*.
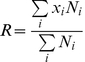
(4)


**Figure 7:**
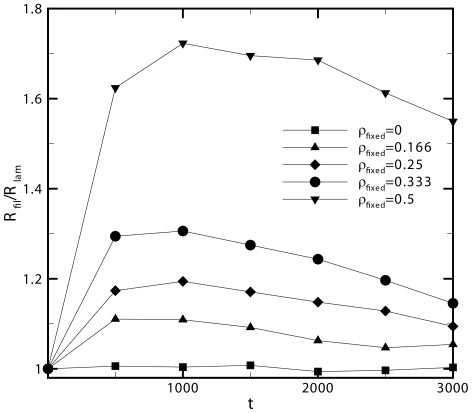
Directionality of actin monomer diffusion as a function of time and concentration. Ratio between the center of mass of particles diffused in the filopodia to that of the lamellipodia as a function of time for different fixed actin filament volume densities. There is a tendency for particles to diffuse towards the filopodia region as a result of the geometry of filaments in each region. This phenomenon is only amplified as the density of fixed actin filaments is increased.

Fig. 7 shows the ratio between the center of mass of particles diffused in the filopodia to that of the lamellipodia as a function of time for different fixed actin filament volume densities using simulation parameters of *ΔL = 5nm* and *D = 5.65 µm^2^/s* in a three dimensional space, *N_Dimension_ = 3* with a movement probability of *T = 1* given that only one particle type is diffusing in the simulation. The general trend seen from these results is that there is an initial peak in the tendency of particles to diffuse into the filopodia region (region with parallel filaments) for all fixed particle densities greater than zero. Subsequently, this peak diminishes over time (ratio decreases towards 1) as particles reach the x-direction extremes and begin to distribute uniformly throughout space.

In addition, it can be seen that as the density of fixed actin filaments is increased, the tendency of particles to diffuse towards the region of parallel filaments is only increased. This is most likely a result of random filaments generating a longer path that must be taken from the center of the simulation box to the left extreme whereas the parallel filaments generate the shortest possible distance that can be taken from the center of the simulation box to the right extreme which is effectively a reduction of dimensionality. Finally, sensitivity analysis was performed on the effect of varying bond angle between filaments in the lamellipodia. Simulations were performed with the original angle of 68±2 degrees as well as 90±2 and 46±2 degrees with a filament volume fraction density of 0.15. We observed the mean ratio of center of mass of particles diffused towards the filopodia to that of those diffused towards the lamellipodia at 68 degrees to fall within one standard deviation of the mean for 90 and 46 degrees. Subsequently, we conclude that the angle between bound filaments in the lamellipodia doesn't directly contribute to the directionality of diffusion at the filopodia/lamellipodia interface. Rather, the aggregation of multiple filament connections, which form a web-like network, results in the impedance of diffusion in any given direction, from which biased diffusion in the direction parallel to the filopodia emerges.

## Methods

### Relating Diffusion Coefficients to ABM Movement Probabilities

Fick's second law relates the effect of diffusion on the concentration field of particles over time [34]. We can express this relationship in terms of the probability of discretized cells being occupied rather than concentration, Eq. (5), by considering the relationship between concentration and the probability of a cell being occupied by an agent, Eq. (6).
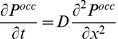
(5)

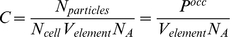
(6)


Note that the variables *P^occ^*, *D, t*, and *x* in Eq. (5) represent probability of finding an occupied cell, diffusion coefficient, time, and position respectively while the variables *C*, *N_particles_*, *N_cell_*, *V_element_*, *N_A_*, and *P^occ^* in Eq. (6) represent concentration, number of particles, number of cells, volume of each element, Avagadro's number and the probability of finding an occupied cell respectively.

The diffusion term in Eq. (5) is a factor dependent on temperature of the solvent, size and shape of the particle, and viscosity of the solvent that quantifies the ratio of Brownian forces to drag forces. Factors such as force and velocity are not explicitly calculated in a simple agent based model and the coarse discretization of space that limits the direction of movement would make such calculations meaningless. Rather, in an agent based model, diffusion can be simulated by assigning a probability of movement to each particle agent. The relation between movement probability and a physically meaningful diffusion coefficient is derived below.

**Figure 8:**
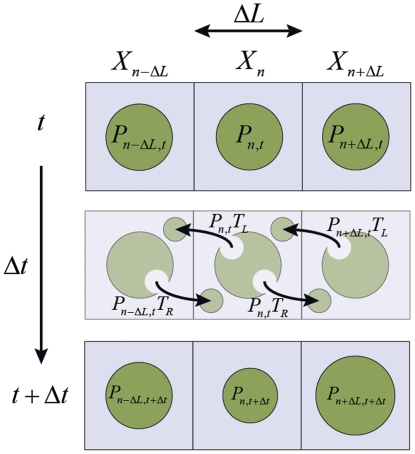
Illustration of movement probabilities in a single dimension. Discretized one-dimensional space with square lattices of length ΔL depicting how probabilities of particles existing in a cell at time *t* combined with movement probabilities result in a change in the probability of a particle occupying a cell at time *t+Δt* as outlined by Eq. 7. Note that the circles in each cell do not represent individual particles; rather they qualitatively represent probabilities of a particle residing in that cell.

We consider a one-dimensional lattice with discretized segments of length *ΔL* as shown in Fig. 8 to derive the relationship between a physical diffusion coefficient and a movement probability to be used in our agent based model. We can define the probability of finding a single particle at position *X_n_* at time *t+Δt* as:
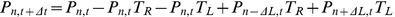
(7)


Where *P_n,t_*, *P_n-ΔL,t_* and *P_n+ΔL,t_* represent the probability of finding the particle at position *X_n_*, *X_n-ΔL_* and *X_n+ ΔL_* respectively at time *t*; T_R_ and T_L_ represent the probability of the particle moving to the right or left respectively. Note that unless otherwise noted, all probability terms represent the probability of the respective cell being occupied. Eq. (7) states that the probability of a particle being found at *X_n_* at time *t+Δt* can be determined based on the probability that the particle was initially in that position and remained there (*first term*) less the probability that the particle started in that position and moved to either the right or left cells (*second and third term*) plus the probability that the particle was initially to the left or right of that cell and moved to the right or left respectively (*fourth and fifth term*).

Taylor expansion of the terms in Eq. (7) as 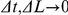 gives the following relationship:
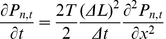
(8)


This assumes that movement probability in both directions are equal (*T_L_+T_R_ = 2T*). Note that the T/2 factor would translate to T/4 and T/6 for two-dimensional and three-dimensional cases respectively. Eq. (8) relates how the transition probability affects the spatial distribution of particles with time, similar to Eq. (5). Thus we can relate diffusion (*D*) to movement probability (*T*), using discretization length (*ΔL*) and time (*Δt*) along with the dimensionality of the environment (*N_dimension_*, either 1, 2 or 3) as previously shown in Eq. (1) for diffusion of a particle on a discrete cubic lattice.

### Crowding effects on Movement Probability

The computational model and analytical solutions described thus far pertain to the diffusion of a single particle in a discretized space. In addition, it is necessary to validate the model behavior in high particle concentrations to ensure that the model behaves in accordance with physical phenomena. Using the same approach used to determine movement probability for a single particle in Eq. (1), we can analytically derive the effective diffusion coefficient for a high concentration, multiple particle system in a stochastic agent based model. Given the same one-dimensional discretized environment from Fig. 8, we can modify Eq. (7) to now incorporate the effect of multiple particles.
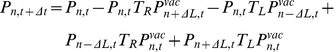
(9)


Where *P^vac^* represents the probability of finding the given cell to be vacant. Eq. (9) states that the probability of a particle being found at *X_n_* at time *t+Δt* can be determined based on the probability that the particle was initially in that position and remained there less the probability that the particle started in that position and moved to either the right or left cells plus the probability that the particle was initially to the left or right of that cell and moved to the right or left respectively if cell *X_n_* was vacant plus the probability of the particle in cell *X_n_* attempting to move to the right or left into an occupied cell resulting in the particle remaining in cell *X_n_*. Taylor series expansion of the time and position varying terms along with the relationship that *P^vac^*  =  *1-P^occ^* gives the solution shown in Eq. (10).
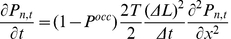
(10)


Note that Eq. (10) is similar to the relationship derived for the single particle concentration field and has the same relationship relating movement probability to diffusion coefficient as the single particle with an additional term related to the probability of cells being occupied by particles. As shown in Eq. (6), this probability of cells being occupied by particles is directly related to the concentration of the system (*C = P^occ^/V_element_N_A_*).

### Model Details

Our agent based model consists of a three-dimensional discretized space that can be bounded or unbounded in which various types of agents diffuse by moving between neighboring cells of cubic shape with a given movement probability, which is equal in all directions and corresponds to the particle's respective diffusion coefficient (Eq. (1)). In this model, we incorporate a von Neumann neighborhood consisting of the six cells orthogonally surrounding an agent in 3D space. Agents in this model can only interact with other agents within their von Neumann neighborhood and can only move in the direction of von Neumann neighborhood cells.

**Figure 9:**
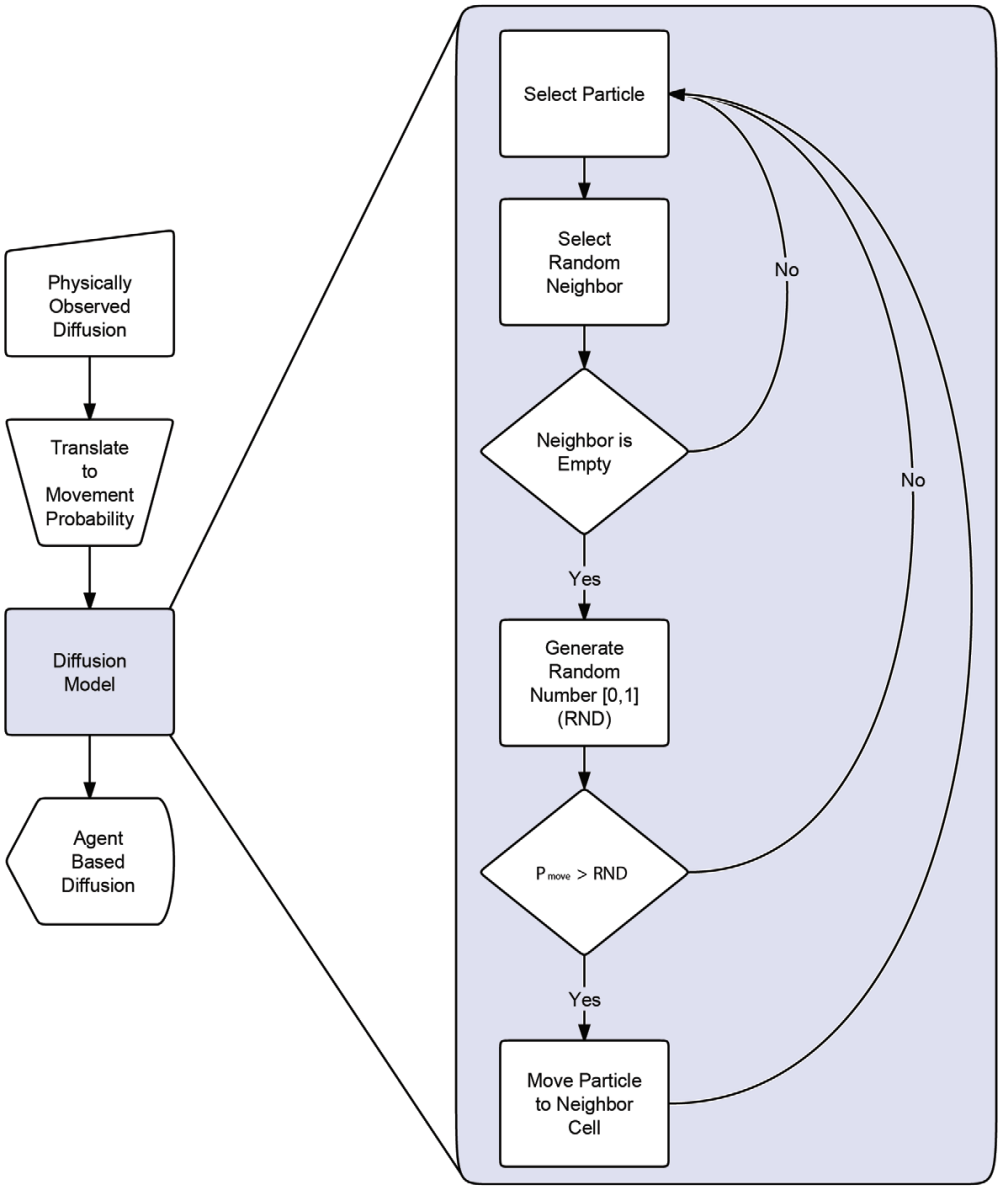
Model validation process. Method for modeling diffusion using physically observed diffusion coefficients (translated to movement probabilities) as an input in an agent based model. Additional details regarding the movement algorithm (specifically the single-neighbor attempt) are illustrated.

At higher concentrations, two methods for particle movement consisting of an *all-neighbor attempt* and a *single-neighbor attempt* algorithm are assessed. These movement methods differ in that an all-neighbor attempt is an intelligent agent movement procedure in which all von-Neumann neighborhoods are searched at random until an empty cell is found for the agent to move to while in the single-neighbor attempt a von Neumann neighbor is selected at random, disregarding whether it is occupied or vacant. If the cell is occupied the movement is rejected and the agent remains in its current cell while movement into a vacant cell is accepted with some probability correlated to the diffusion coefficient. In this second method, rejected movements represent a collision between two particles resulting in both particles remaining in their respective cells. Fig. 9 also shows the process for single-neighbor attempt movements.

**Figure 10:**
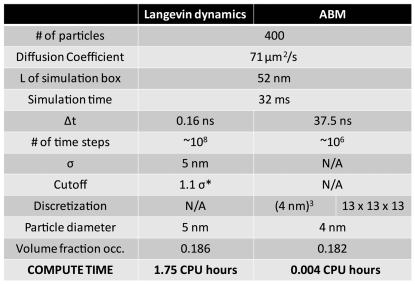
Comparison of computational costs between agent based and Langevin dynamics methods. Benchmark showing compute time between a Langevin dynamics simulation and an agent based model of 400 particles diffusing in the same size simulation box and both simulations having the same fraction of volume occupied. The agent based model used a discretized simulation box of 13×13×13 with cubic cells of 4 nm in length. The Langevin dynamics model incorporated the *Cichocki and Hinsen* method [36] for simulating hard-spheres and utilized further optimizations such as neighbor lists to maximize computational efficiency. *The Langevin dynamics simulation cutoff was set lower than typical to simulate hard spheres to match that of the agent based model. Typical Langevin dynamics models use higher cutoffs resulting in additional computation time.

The model was developed using object oriented FORTRAN to maximize computational efficiency. Agent based modeling benefits significantly from object oriented programming since the concept of an object is similar to the concept of an agent. Moreover, agent based modeling is very computationally efficient for large systems and long time scales when compared to modeling techniques such as Langevin dynamics as shown in Fig. 10 at the cost of reduced spatial and temporal detail. Additionally, the discrete nature of the model makes it an ideal candidate for parallelization and distributed computing, resulting in further computational efficiency [35].
